# Selenylated Imidazo [1,2-*a*]pyridine Induces Apoptosis and Oxidative Stress in 2D and 3D Models of Colon Cancer Cells

**DOI:** 10.3390/ph16060814

**Published:** 2023-05-30

**Authors:** Giovana Bicudo Gomes, Claudia Stutz Zubieta, Jhefferson dos Santos Guilhermi, Mônica Cristina Toffoli-Kadri, Adilson Beatriz, Jamal Rafique, Eduardo Benedetti Parisotto, Sumbal Saba, Renata Trentin Perdomo

**Affiliations:** 1 Postgraduate Course in Pharmaceutical Sciences, Federal University of Mato Grosso do Sul (UFMS), Campo Grande 79070-900, Brazil; giovana.bicudo@ufms.br (G.B.G.); claudia.stutz@gmail.com (C.S.Z.); monica.kadri@ufms.br (M.C.T.-K.); eduardo.parisotto@ufms.br (E.B.P.); 2 Instituto de Química (IQ), Universidade Federal de Goiás (UFG), Goiania 74690-900, Brazil; jheffersonguilhermi@discente.ufg.br (J.d.S.G.); or jamal.rafique@ufms.br (J.R.); 3 Laboratory of Synthesis and Transformation of Organic Molecules (SINTMOL), Institute of Chemistry (INQUI), Federal University of Mato Grosso do Sul (UFMS), Campo Grande 79074-460, Brazil; adilson.beatriz@ufms.br

**Keywords:** ROS generation, oxidative damage, cytotoxicity, cancer, cell death, selenium

## Abstract

Colon cancer incidence rates are increasing annually, a scenario aggravated by genetic and epigenetic alterations that promote drug resistance. Recent studies showed that novel synthetic selenium compounds are more efficient and less toxic than conventional drugs, demonstrating biocompatibility and pro-oxidant effects on tumor cells. This study aimed to investigate the cytotoxic effect of MRK-107, an imidazo [1,2- *a*]pyridine derivative, in 2D and 3D cell culture models of colon cancer (Caco-2 and HT-29). Sulforhodamine B results revealed a GI50 of 2.4 µM for Caco-2, 1.1 µM for HT-29, and 22.19 µM for NIH/3T3 in 2D cultures after 48 h of treatment. Cell recovery, migration, clonogenic, and Ki-67 results corroborated that MRK-107 inhibits cell proliferation and prevents cell regeneration and metastatic transition by selectively reducing migratory and clonogenic capacity; non-tumor cells (NIH/3T3) re-established proliferation in less than 18 h. The oxidative stress markers DCFH-DA and TBARS revealed increased ROS generation and oxidative damage. Caspases-3/7 are activated and induce apoptosis as the main mode of cell death in both cell models, as assessed by annexin V-FITC and acridine orange/ethidium bromide staining. MRK-107 is a selective, redox-active compound with pro-oxidant and pro-apoptotic properties and the capacity to activate antiproliferative pathways, showing promise in anticancer drug research.

## 1. Introduction

More than 1.9 million cases of colon cancer are detected each year worldwide, and, for half of these cases, the outcome is death [1,2]. Colon cancer patients may develop drug resistance, resulting in reduced treatment efficacy and the need for chemotherapy [3]. Tumor biological behavior is evaluated through pathological characteristics and molecular markers that indicate the prognostic value of colorectal cancer, assisting in the search for new drugs with more effective responses and lower toxicity [4]

Organoselenium compounds have interesting anti-cancer properties, attributed to their biocompatibility and pro-oxidant effects on tumor cells [5,6,7,8,9]. These compounds have been shown to inhibit colon tumor growth in HT-29 and Caco-2 cells, but their efficacy still needs improvement [10,11]. Selenylated imidazo [1,2- *a*]pyridine, a synthetic organoselenium compound, causes reactive oxygen species (ROS)-induced oxidative damage, which may promote cancer cell death [12,13,14,15]; however, the synthetic compound needs to be modulated by including functional groups in the selenium-bound portion for enhanced selectivity toward cancer cells [16,17,18,19].

Three-dimensional (3D) cell spheroid models are known to mimic the tumor microenvironment, having been successfully used for drug screening and the study of tumor growth, proliferation, invasion, micrometastasis, and immune cell interactions [20,21]. Another advantage is the reduction in animal experimentation, which is beneficial for ethical and scientific reasons, given the discrepancies in responses between species and the difficulty in extrapolating results to humans [22].

Thus, in view of our continuing interest in biologically relevant organoselenium compounds and functionalization of imidazo [1,2- *a*]pyridines [23,24,25,26,27,28,29,30], this study aimed to investigate the effect of MRK-107 in two-dimensional (2D) and 3D (spheroid) models of colon carcinoma, with a focus on induction of cell death by proliferative, inflammatory, apoptotic, or multiple oxidative stress-related pathways.

## 2. Results

### 2.1. MRK-107 Selectively Decreased Caco-2 and HT-29 Viability in Monolayers, Affording Colony Reduction and Impaired Healing/Recovery

**Figure 1:**
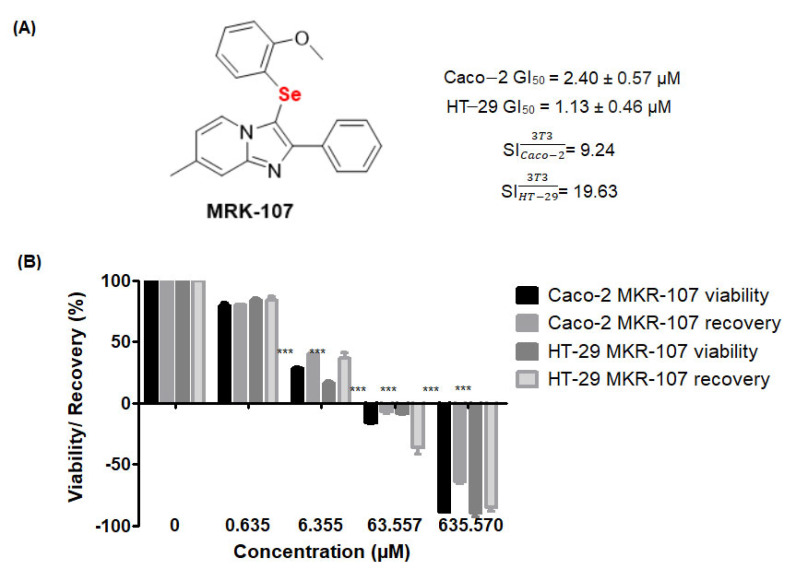
Structure of MRK-107, a selenylated imidazo [1,2-*a*]pyridine derivative, and its effect on cell viability at different concentrations (0.6–635.57 µM). (**A**) Structure of MRK-107, GI_50_ against Caco-2 and HT-29, and selectivity indices (SI). (**B**) Viability and recovery of Caco-2 and HT-29 cells after 48 h of treatment with MRK-107; *** *p* < 0.001. Effects on viability were significant at 6.355, 63.55, and 635.57 µM MRK-107. A recovery of less than 50% was deemed irrelevant.

The effect of MRK-107 (0.635–635.570 µM) on the viability and recovery of Caco-2 and HT-29 cells was assessed by the sulforhodamine B (SRB) assay (Figure 1). MRK-107 (Figure 1A) significantly reduced the viability of Caco-2 and HT-29 cells after 48 h at concentrations of 6.355, 63.55, and 635.57 µM (Figure 1B), with a half-maximal growth inhibitory concentration (GI_50_) of 2.40 µM for Caco-2 and 1.13 µM for HT-29.

**Figure 2:**
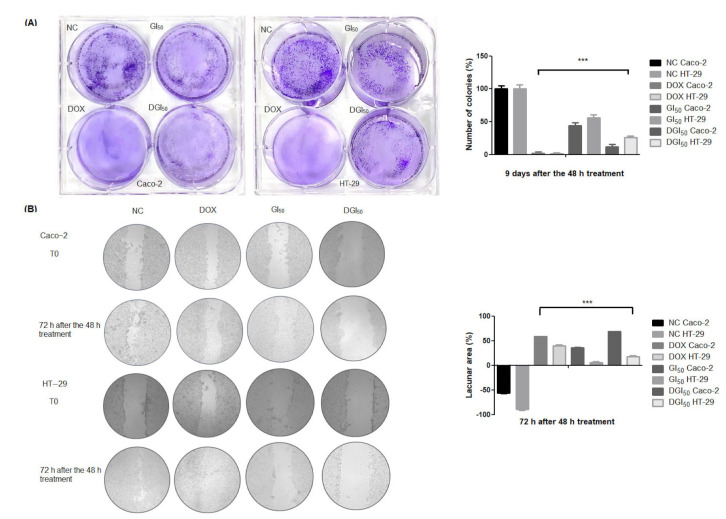
Cytotoxicity and proliferation assays of colon cancer cells treated with MRK-107 and their quantitative analysis. (**A**) Colony formation in the negative control (NC) and Caco-2 and HT-29 cells at 48 h after treatment with MRK-107 or doxorubicin (DOX, positive control) and their quantitative analysis. (**B**) Cell migration of Caco-2 and HT-29 and their quantitative analysis; *** *p* < 0.001 compared with NC. 100× magnification.

Furthermore, exposure of Caco-2 and HT-29 cells to GI_50_ and double the GI_50_ (DGI_50_) induced a significant decrease in clone formation (Figure 2A), as well as in migration/healing (Figure 2B). These results suggest that, after 48 h of exposure, the cytotoxic effect of MRK-107 is independent of incubation time and HT-29 cells are more sensitive to long-term MRK-107 exposure than Caco-2 cells. MRK-107 at DGI_50_ was effective in inhibiting migration. HT-29 cells exhibited reduced individual migration, attributed to the phenotype of the cell line.

### 2.2. MRK-107 Reduces Proliferation

The Ki-67 index was determined after treatment of Caco-2 and HT-29 cells with MRK-107 at GI_50_ and DGI_50_. The proliferation index was higher in untreated cells, which translates clinically into hyperproliferation, low tumor differentiation, and worse prognosis. A reduction of Ki-67 expression by at least 14% represents a loss of proliferative capacity, having a direct impact on cell cycle activity. MRK-107 afforded reductions of 50% and 45% in Caco-2 and HT-29 proliferation, respectively (Table 1).

### 2.3. MRK-107 Causes Oxidative Stress, Generates Protein Damage, and Ruptures DNA in Caco-2 and HT-29 Cells in Monolayer and Spheroid Cultures

**Figure 3:**
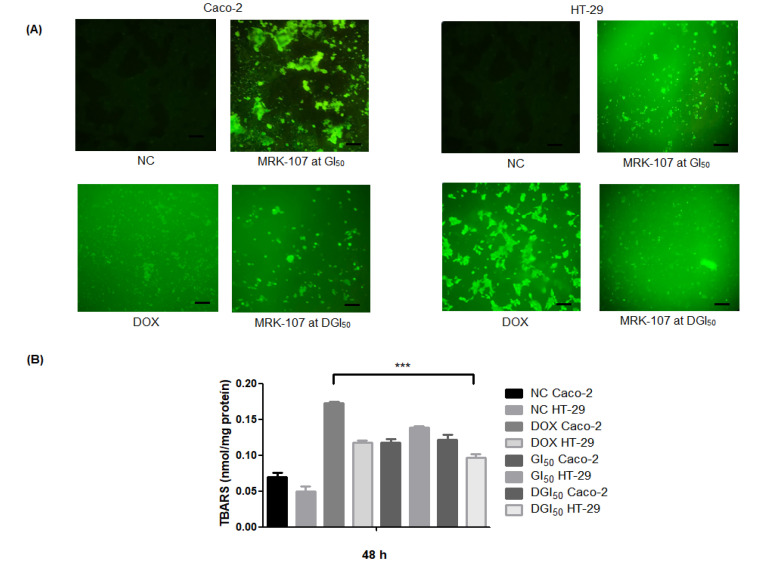
Oxidative stress in colon cancer cells in response to MRK-107 treatment. (**A**) DCFH-DA staining in the negative control (NC) and Caco-2 and HT-29 cells at 48 h after treatment with MRK-107 or doxorubicin (DOX, positive control). (**B**) TBARS content of Caco-2 and HT-29 cells at 48 h after treatment, as assessed by spectrophotometry. *** *p* < 0.001 compared with NC. Scale bar = 20 µm.

Oxidative stress caused by MRK-107 treatment was quantified using 2,7-dichlorofluorescein diacetate (DCFH-DA). The negative control showed low fluorescence intensities compared with the positive control and MRK-107-treated samples (Figure 3A). In MRK-107 treatments, fluorescence was observed in both cell lines. The TBARS content of Caco-2 remained constant even under increasing MRK-107 concentrations; in HT-29, however, there was a reduction of oxidative damage at the highest MRK-107 concentration, although TBARS content was high (Figure 3B).

### 2.4. MRK-107 Activates Caspases-3/7 in Caco-2 and HT-29 Cells in Monolayer and Spheroid Cultures

**Figure 4:**
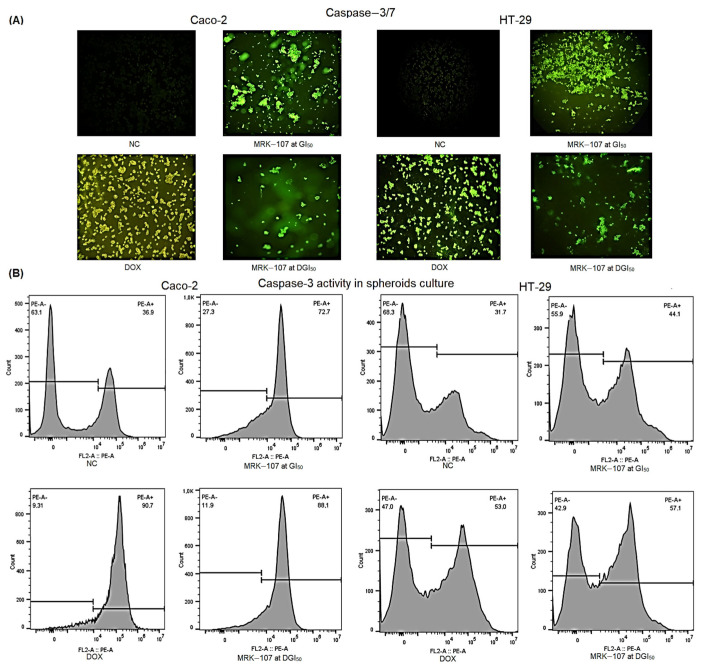
Caspase-3/7 activities in colon cancer cells treated with MRK-107. (**A**) Fluorescent signals of the negative control (NC) and Caco-2 and HT-29 cells at 24 h after treatment with MRK-107 or doxorubicin (DOX, positive control), as assessed using the CellEvent kit. (**B**) Caspase-3 activity in 3D cultures of Caco-2 and HT-29 at 24 h after treatment, as assessed by flow cytometry. Scale bar = 20 µm.

The effect of MRK-107 on caspase-3/7 activities was determined to confirm the flow cytometric results of spheroid cultures. The apoptotic cell death marker CellEvent (Figure 4A) and PE Rabbit Anti-Active Caspase-3 (Figure 4B) were used for flow cytometry assays. MRK-107 increased caspase-3/7 activities after 24 h of treatment in both cell lines at both concentrations.

### 2.5. MRK-107 Induces Apoptosis of Caco-2 and HT-29 Cells

**Figure 5:**
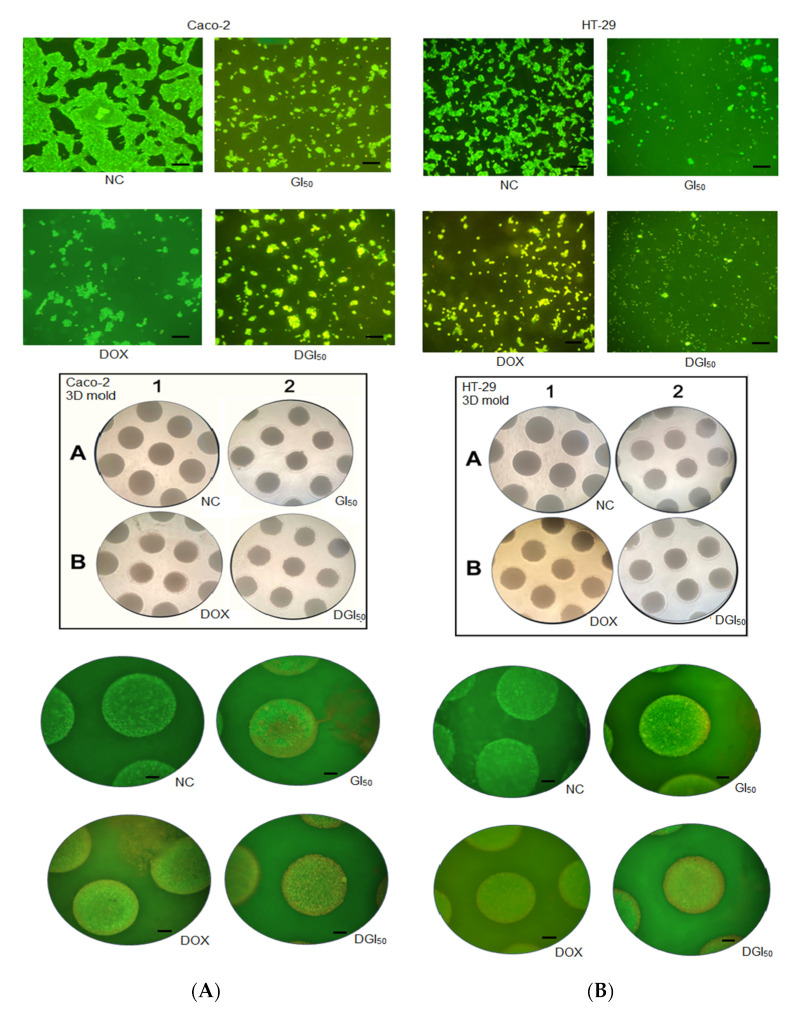
Cell death in response to MRK-107 in 2D and 3D cultures of Caco-2 and HT-29 at 48 h after treatment, as assessed using acridine orange and ethidium bromide staining. In the negative control (NC, normal cells), note the circular nucleus uniformly distributed in the center of cells. MRK-107 and doxorubicin (DOX, positive control) treatments resulted in early and late apoptotic cells, with the nucleus showing yellowish-green fluorescence by acridine orange staining. (**A**) Acridine orange/ethidium bromide staining of 2D and 3D Caco-2 cell cultures and the cells after 48 h of treatment in spheroid molds. (**B**) Acridine orange/ethidium bromide staining of 2D and 3D HT-29 cell cultures and the cells after 48 h of treatment in spheroid molds. Scale bar = 100 µm.

**Figure 6:**
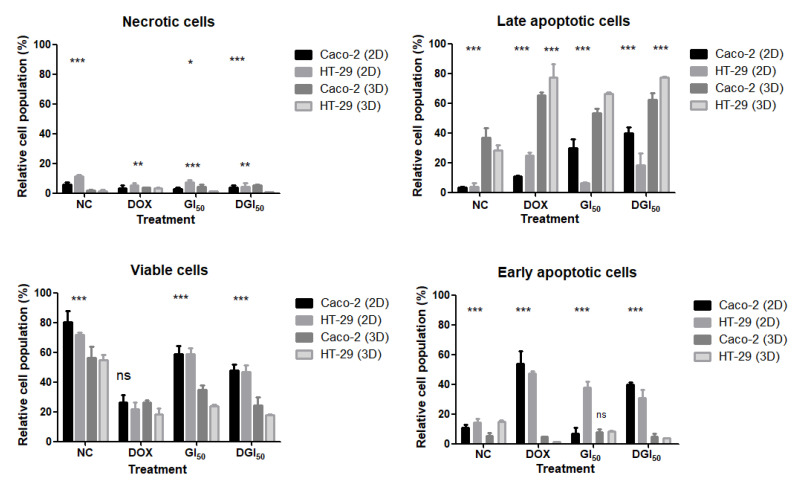
Annexin V and propidium iodide results of negative control (NC) and Caco-2 and HT-29 cells in 2D and 3D models at 48 h after treatment with MRK-107 or doxorubicin (DOX, positive control). Fluorescent population of necrotic, viable, early apoptotic, and late apoptotic cells in 2D and 3D cultures, as evaluated using flow cytometry. Asterisks indicate significant differences between 2D and 3D models; *** *p* < 0.001, ** *p* < 0.01 and * *p* < 0.05; ns—not significant.

MRK-107-induced cell death of Caco-2 and HT-29 was evaluated using differential fluorescent staining with acridine orange and ethidium bromide. MRK-107 was used at the GI_50_ for Caco-2 (2.40 µM) and HT-29 (1.13 µM), as well as at the DGI_50_, in 2D and 3D cultures. In monolayer cultures treated with MRK-107, there was a dose-dependent loss of anchorage and presence of early-stage apoptotic cells, as evidenced by the yellow-green fluorescence of the cell nucleus. Stains were located asymmetrically within cells. With increasing MRK-107 concentration and treatment duration, the number of early-stage apoptotic cells increased. Late-stage apoptotic cells, characterized by concentrated and asymmetrically located orange nuclear staining, were also detected (Figure 5 and Figure 6). Necrotic cells increased in volume and showed orange-red fluorescence.

The 3D culture showed higher staining definition, with signs of advanced apoptosis, vacuole formation, and textural changes in cell interactions after treatment with MRK-107. The percentages of viable, early apoptotic, late apoptotic, and necrotic Caco-2 and HT-29 cells are shown in Figure 6. The annexin V-FITC/propidium iodide assay was used to determine the cell death profile of 2D and 3D cultures. Caco-2 and HT-29 cells showed reduced viability, with significant differences between cell lines and culture types. MRK-107 toxicity was found to be dose-dependent. The 2D culture had high peaks of early cell death, whereas the 3D culture had mainly late apoptosis peaks. Overall, there was a low presence of necrotic cells, being lowest in the HT-29 3D model. Regardless of the cell model used, MRK-107 demonstrated pro-apoptotic action at both concentrations, as assessed using the annexin V-FITC detection kit.

## 3. Discussion

The numerous mutations of colon cancer cells contribute to the poor prognosis of the disease. The incidence and mortality rates of colon cancer are predicted to increase [31], underscoring the importance of improving the survival response, whether through diagnostic markers or chemotherapy [32]. Selenium has been widely evaluated for its chemopreventive potential, but its toxic effects are a cause for concern and its use in food and supplementation is limited [33,34]. New molecules, such as aryl selenoester, may enhance treatment selectivity, thereby minimizing side effects [35]. The selectivity indices of MRK-107 for Caco-2 (9.24) and HT-29 (19.63) were excellent, demonstrating that the compound can be used without altering the proliferative capacity of normal cells. The cell inhibition concentrations of MRK-107 were 2.40 µM for Caco-2 and 1.13 µM for HT-29, representing high anticancer activity [36]. Both cell lines originate from the same region of the colon but have specific characteristics in terms of transepithelial/endothelial resistance [37]. For instance, the physiological barrier of Caco-2 cells is four times more resistant than that of HT-29 cells. This fact might be related to the two-times higher GI_50_ for Caco-2 as compared with HT-29.

A previous study showed that compounds with a similar chemical structure to MRK-107 exhibited moderate activity in vitro and in vivo [38]. Other selenium compounds were shown to have potent anticancer activity against colon cancer cells (GI_50_ = 3.9 µM) but no selectivity (GI_50_ = 3.5 µM for fibroblasts); structural modification with the aim of increasing selectivity resulted in moderate anticancer activity [39]. Chemical modification of MRK-107 with 2-methoxyphenyl seems to be sufficient to confer polarity and electrophilicity when binding to selenium; organoselenium compounds can act as redox modulators, showing preference for malignant cells and protecting normal cells [40]. The methoxy group reflects a greater possibility of oxidative reactions; thus, MRK-107 has more intense lipoperoxidation because it generates damage to the thiol groups [41]. Another ally in the structure–activity relationship of selenium organocompounds is methyl attached to the aromatic ring, which affects the mitochondrial membrane potential and facilitates the apoptotic process [42]. Cytotoxic effects during treatment may be followed by recovery of proliferation or permanent damage, which leads to cell death [43]. Previous research with selenium compounds showed decreased migration and invasion of cancer cells. MRK-107 caused permanent damage, leading to inefficient proliferation recovery, as evaluated using standards that disregard <50% cell growth; these findings demonstrate that MRK-107 also prevents new clone formation in the long term [44,45]. The proliferation index of cancer cells was 2.4–3, demonstrating concentration-dependent cellular quiescence [46]. MRK-107 reduced the proliferative index by 29% at GI_50_ and by at least 45% at DGI_50_. The decrease in cell proliferation and induction of cell death by apoptosis was proportional to the inhibition of cell division progression. Selenium compounds were found to increase the proportion of Caco-2 and HT-29 cells in the sub-G1 phase by blocking the progression to the S phase [47]. Some cytotoxic treatments involving selenium, such as MRK-107, are dependent on caspases to generate cytotoxicity [48].

MRK-107 is a selenoether prone to oxidize and release redox-active compounds [49]. A possible mechanism of action of MRK-107 through redox shift toward oxidation would involve the cross-linking of protein thiol groups and the promotion of ROS generation, propitiating lipid peroxidation events, DNA damage, mitochondrial respiratory chain destruction, and protein modifications [50]. The increased fluorescence of DCFH-DA is interpreted primarily as resulting from the increased production of free radicals mediated by hydrogen peroxide, a product of nitric oxide and superoxide reactions [51]. In the 2D model, we detected the most expressive oxidative stress in HT-29 cells. In the 3D model, peroxidation was also highest in HT-29, but values decreased at the highest concentration. In Caco-2 cells, DCFH-DA fluorescence was stronger in the 2D model. In the 3D model, oxidative damage reached a plateau at the highest doses, thereby indicating a great possibility of damage to cell membranes, proteins, amino acids, and DNA [52].

Oxidative stress in the tumor microenvironment has a pro-inflammatory action directed at enhancing antitumor immunity and inducing cell death signaling pathways [53]. There are several intermediate forms of cell death with apoptotic and necrotic morphological features. It is possible to gain insight into such processes by analyzing membrane permeability [54]. Caspases−3 and −7 have an effector function and act downstream of both intrinsic and extrinsic apoptotic pathways [55]. MRK-107 produced expressive results in both 2D and 3D culture models, but activation was more intense in Caco-2 cells upon doxorubicin (DOX) treatment. It is known that the cytotoxic action of compounds commonly differs between 2D and 3D models. This discrepancy is due to the organizational behavior of tumor cells, which are prone to produce necrotic regions with poor vascularization and hypoxic conditions, creating a therapeutic gradient. Such a gradient can be observed by differential staining with acridine orange and ethidium bromide, which reveals the loss of cellular arrangements in three different zones and of variable sensitivity [56].

The main type of cell death was early apoptosis in the 2D model and late apoptosis in the 3D model. Apoptosis susceptibility is determined by interactions between cells, the cell matrix, and ROS production/signaling. In the MRK-107 treatment, it was possible to observe cell deformation and vesicle formation at GI_50_. At DGI_50_, the apoptotic effect was evident. A better understanding of ROS generation by cells that constitute the tumor microenvironment is critical to improve therapeutic options and clinical outcomes [57]. The 2D culture overestimates MRK-107 toxicity, but the 3D model achieved more than 50% toxicity at low MRK-107 concentrations. Treatment reduced cell anchorage, characterizing anoikis. As this process depends on activation of oxidation by the detached cell [58], MRK-107 is believed to act via DCFH-DA, TBARS, and annexin V.

Recreating appropriate epithelial–extracellular matrix interactions is a challenge and we are moving toward more robust analyses [59]. Our 3D models supported the formation of an epithelium with a tissue layer similar to the stroma, and MRK-107 was able to disrupt this construction. The Notch proliferation pathway is overexpressed in colon cancer and acts on cell interactions; however, MRK-107 appears to precisely reduce dysregulated expression due to the loss of cell–cell interactions [60]. Other signaling pathways related to reduced cell growth, reduced migration, and increased apoptosis, such as PI3K/Akt, MAPK/ERK, and Wnt/β-catenin, might have been inhibited by MRK-107 treatment [61]. Selenium compounds can inhibit β-catenin, a molecule that generates drug resistance and whose inhibition increases cytotoxicity [62]. In colon cancer, the methylation of histones H3 in K9 generates resistance to the 5-flu chemotherapy, but selenium compounds are associated with the inhibition of these histones and thus the expression of Fas increases and stimulates apoptosis [63,64]. Thus, MRK-107 acts through a combined mechanism based on the accumulation of oxidative stress and the breakdown of signaling molecules necessary for the proliferative activity of cancer cells. Alteration of the membrane potential initiates cytochrome c efflux, which consequently activates caspases-3 and -9. These effects are similar to those of imidazole-derived compounds used to treat colon cancer [65]. Moreover, selenium is considered a promising anticancer agent with lower toxicity, higher bioavailability, and a broad spectrum of biological activities, including antioxidant activity, which allows modulation of aberrant proliferation pathways and activates inflammation, apoptosis, and other cell death-related pathways [66,67].

## 4. Materials and Methods

### 4.1. MRK-107 Synthesis

The selenylated imidazo [1,2-*a*]pyridine MRK-107 was synthesized by reaction of imidazo [1,2-*a*]pyridine with diselenide via C(sp^2^)–H bond selenylation, as previously reported by us [68,69,70,71].

### 4.2. Cell Lines and Treatments

Caco-2 (HTB-37) and HT-29 (HTB-38) cell lines were purchased from American Type Culture Collection (ATCC^®^, Manassas, VA, USA). Both cell lines were grown in monolayer under the following conditions: 100% humidity, 37 °C, and 95% air, and 5% CO_2_ atmosphere. Cells were grown in RPMI 1640 (Gibco (Waltham, MA, USA), Life Technologies, Austin, TX, USA) supplemented with 10% fetal bovine serum (Gibco, Life Technologies, USA). Three-dimensional model cells were grown in Dulbecco’s modified Eagle’s medium (DMEM) (Gibco, Life Technologies, USA) supplemented with 10% fetal bovine serum (Gibco, Life Technologies, USA). Both complete growth media were also supplemented with 50 IU/mL penicillin/streptomycin (Gibco, Life Technologies, USA). Cells were collected after the third passage at the logarithmic growth phase. Trypan blue (Sigma–Aldrich, Burlington, MA, USA) was used to count live cells. MRK-107 was synthesized, and a stock solution of MRK-107 was prepared in dimethyl sulfoxide (DMSO) (Sigma–Aldrich, USA). Cells were exposed to MRK-107 and DOX (positive control) (Eurofarma, Brazil) for 48 h.

### 4.3. Cytotoxicity Assay

The cytotoxicity of MRK-107 and DOX was determined using SRB (Sigma–Aldrich, USA). Caco-2, HT-29, and NIH/3T3 were seeded in 96-well plates at a density of 3 × 10^5^ cells/mL per well. After overnight culture to reach logarithmic growth, MRK-107 (0.635–635.57 µM) and DOX (0.046–48 µM) were added, and cells were incubated for 48 h. The cells were fixed with 20% trichloroacetic acid and, after rinsing and drying, stained with 0.1% SRB. The controls were DOX (positive control), negative control, sample blank, and reading at time zero (before treatment). The optical density was obtained at 540 nm by using a 96-well microplate reader (SpectraMax 190, Molecular Devices, Silicon Valley, CA, USA) [72]. The results are the mean of three independent experiments (*n* = 3). GraphPad Prism software was used to calculate the concentration that reduces the growth of treated cells by 50% with respect to untreated controls (GI_50_) [73].

### 4.4. Selectivity Assay

The selectivity index was calculated as the ratio of the GI_50_ of the test compound for non-tumor cell lines (3T3/NIH) to the GI_50_ for cancer cells (Caco-2 and HT-29) [74].

### 4.5. Assay

To study the recovery in cell proliferation after drug removal, we first treated Caco-2 and HT-29 cells as described above (Section 4.3). Then, the medium was removed and replaced with fresh medium without the drug. The rate of cell recovery was assessed over the subsequent 48 h by the SRB assay. Cell viability results were compared between the treatment plate and the plate that was washed and kept in culture after treatment [75].

### 4.6. Clonogenic Assay

Caco-2 and HT-29 cells were plated in 6-well plates at a density of 500 cells/mL and treated for 48 h with or without MRK-107 at GI_50_ or DGI_50_. DOX was used at 3.68 µM. After incubation, the medium was removed and replaced with fresh drug-free medium. After 9 days, the supernatant was removed and colonies were fixed with 10% formaldehyde for 15 min, stained with 0.5% crystal violet for another 15 min, and counted under an inverted microscope [76]. The number of colonies was counted using ImageJ software (National Institutes of Health, USA) [77].

### 4.7. Wound-Healing Assay

Caco-2 and HT-29 cells were seeded at a density of 2 × 10^5^ cells/mL in 24-well plates. When cells reached 80% confluence, the supernatant was discarded and the cell monolayer was scraped to create a straight-line gap. Treatments were as follows: MRK-107 at GI_50_ and DGI_50_, negative control, and DOX at 3.68 µM. The results were analyzed after 48 h of incubation by ImageJ program (National Institutes of Health, USA) [78].

### 4.8. Ki-67 Staining

Caco-2 and HT-29 were plated at a density of 5 × 10^5^ cells/mL and incubated with MRK-107 at GI_50_ or DGI_50_, a negative control solution, or DOX at 3.68 µM for 48 h. Cells were trypsinized and centrifuged. The supernatant was discarded, and cells received the addition of 100 µL of PBS with pre-diluted Ki-67 antibody [79]. Plates were incubated at 4 °C in the dark for 30 min and then centrifuged and washed with PBS. Readings were taken using a CytoFLEX cytometer (Beckman Coulter, Brea, CA, USA). Data were analyzed using FlowJo software version 10.8 (BD Life Sciences, Franklin Lakes, NJ, USA).

### 4.9. DCFH-DA

Caco-2 and HT-29 cells were seeded at a density of 5 × 10^5^ cells/mL and treated with MRK-107 at GI_50_ or DGI_50_, a negative control solution, or DOX at 3.68 µM for 48 h. Staining was performed using 5 µL of 2′,7′-dichlorofluorescein diacetate (Sigma–Aldrich, USA) diluted in DMSO [80]. Stained cells were observed under a fluorescence microscope.

### 4.10. Spheroid Formation

For spheroid formation, a suspension containing 2 × 10^6^ cells in 120 µL of agarose-supplemented DMEM was pipetted into micromolds (MicroTissues^®^ 3D Petri Dish^®^, Sigma–Aldrich) and incubated at 37 °C in a humidified atmosphere with 5% CO_2_. Caco-2 and HT-29 cells were used. Treatments were as follows: MRK-107 at GI_50_ and DGI_50_ (1.13 and 2.26 µM for Caco-2 and 2.4 and 4.8 µM for HT-29, respectively), DOX at 3.68 µM, and untreated spheroids [81]. The proportion of cell aggregation was monitored at different time points by light microscopy The collection of spheroids for testing was carried out with pipettes and cell dissociation was performed with enzymatic action (0.25% trypsin solution) [82].

### 4.11. TBARS Assay

Lipid peroxidation levels were determined spectrophotometrically at 535 nm in the spheroid homogenate by the thiobarbituric acid-reactive substances (TBARS) method [83]. TBARS values were calculated using a molar extinction coefficient of 153 mM/cm. The absorbance of a pink chromophore was measured in triplicate, and values are expressed as nmol TBARS/mg protein.

### 4.12. Caspases-3/7

Caspase-3/7 activities were detected using the CellEvent™ Caspase-3/7 Green Detection Reagent (Life Technologies). Caco-2 and HT-29 cells were seeded (1 × 10^5^ cells/mL) in 6-well plates and treated for 48 h. After incubation, cells were labeled with 100 µL of the reagent and observed under a fluorescence microscope [84]. For spheroids, labeling was performed using the PE Rabbit Anti-Active Caspase-3 kit (BD Pharmingen™, San Diego, CA, USA). Caco-2 and HT-29 (5 × 10^5^ cells/well) cells seeded on 3D micromolds were treated, dissociated, and the pellet fixed, permeabilized, incubated with 20 μL of the antibody, and analyzed using FlowJo software version 10.8 (BD Life Sciences, USA).

### 4.13. Differential Staining

Spheroids were stained with a solution of acridine orange (100 µg/mL, Sigma–Aldrich, USA) and ethidium bromide (100 µg/mL, Invitrogen, USA) at a final concentration of 1 µg/mL. Immediately after incubation, cells were observed in the dark under a fluorescence microscope (Olympus BX41) using UV excitation [85]. Images were analyzed using ImageJ software (National Institutes of Health, USA) to determine the fluorescence intensity of the two dyes, expressed as percentage of viable (green) and non-viable (red) cells.

### 4.14. Annexin V

Apoptotic cell death in Caco-2 and HT-29 cell cultures was determined using the Annexin-V FITC kit (Sigma Aldrich, St. Louis, USA). Briefly, a suspension containing 5 × 10^5^ cells/mL was seeded in 6-well plates or 3D molds, the supernatant was discarded and washed with PBS, and the cells were resuspended in a binding buffer and incubated with 5 µL of FITC-conjugated annexin-V and, after 15 min, 10 µL of propidium iodide solution (1 mg/mL) at room temperature for 10 min in the dark [86]. Immediately after incubation, cells were analyzed using a flow cytometer (CytoFLEX, Beckham Coulter). Data were analyzed using FlowJo software version 10.8 (BD Life Sciences, USA).

### 4.15. Statistical Analysis

Data from cytotoxicity assays were analyzed using one-way analysis of variance. The normality of continuous variables was confirmed by Tukey’s test using GraphPad Prism software version 5.0 (GraphPad, Inc., La Jolla, CA, USA). Significant differences were accepted at *p* < 0.05. Results are presented as mean ± standard deviation.

## 5. Conclusions

In view of the importance of epigenetic mechanisms in colorectal cancer progression and the possibility of reversal through the application of appropriate drugs, it is concluded that MRK-107, a selenylated imidazo [1,2-*a*]pyridine, is a good candidate in the search for improved tumor therapies, with potential as combination therapy or an enhanced pharmaceutical formulation. The 3D model exhibited improved morphological features, and MRK-107 had considerable cytotoxic action in this model despite physiological barriers. MRK-107 is redox-active, pro-oxidant, pro-inflammatory, pro-apoptotic, and capable of activating additional antiproliferative pathways.

## Figures and Tables

**Table 1:** Cell proliferation index of Caco-2 and HT-29 at 48 h after treatment with MRK-107.

Cell Line	Treatment	Proliferation Index
Caco-2	Negative control	2.67 ± 0.25
	Doxorubicin	1.65 ± 0.27a
	GI_50_	1.32 ± 0.15a
HT-29	DGI_50_	1.25 ± 0.14a
	Negative control	2.41 ± 0.22a
	Doxorubicin	1.44 ± 0.19a
	GI_50_	1.73 ± 0.27a
	DGI_50_	1.33 ± 0.13a

G1_50_, MRK-107 concentration causing 50% cell growth inhibition; DG1_50_, double the GI_50_. Means followed by lowercase letters indicate significant differences at *p* < 0.001 in relation to the negative control.

## Data Availability

Not applicable.
